# Viruses to fight other viruses: the influenza vaccine case

**DOI:** 10.15252/emmm.202012059

**Published:** 2020-04-22

**Authors:** Jose A Bengoechea

**Affiliations:** ^1^ Wellcome‐Wolfson Institute for Experimental Medicine School of Medicine Dentistry and Biomedical Sciences Queen's University Belfast Belfast UK

**Keywords:** Immunology, Microbiology, Virology & Host Pathogen Interaction

## Abstract

Undoubtedly, vaccination is one of the health interventions showing major impact on humankind. Vaccines remain one of the most effective and safest ways to tackle infections. The current coronavirus pandemic is not an exception, and we all hope that ongoing international efforts will succeed in developing a vaccine soon. In this scenario, the present work published in this edition of *EMBO Molecular Medicine* by Demminger and colleagues (Demminger *et al*, 2020) is timeliness to exemplify the steps needed to develop effective vaccines.

## Graphical abstract

When vaccination against a virus uses another virus or the tale of AAV‐mediated vaccines against influenza infections, in this issue of *EMBO Molecular Medicine*.

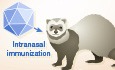

This study focuses on influenza, a seasonal pandemic with major annual impact on the health systems worldwide. This infection is associated with high mortality and morbidity in very young and old individuals. Stories in the recent news have highlighted the unusual deadly influenza pandemic lasting from 1918 to 1920, the so‐called *Spanish flu*, highlighting the devastating impact of these viral infections.

The current influenza prophylaxis is based on immunization with trivalent or quadrivalent influenza vaccines containing antigens from influenza A and B viruses. However, this vaccine is mostly strain specific and the process to produce it is time‐consuming. In addition, evidence indicates it does not provide protection against emerging zoonotic influenza A virus strains. Overall, the need to develop a novel vaccine conferring protection as broad as possible is widely acknowledged.

One observation key for this work is the discovery of protective antibodies against the surface glycoprotein hemagglutinin (HA) acting *via* interference with viral replication or *via* Fc‐receptor‐mediated cellular cytoxicity (DiLillo *et al*, 2016; Wu & Wilson, 2017). Demminger and colleagues decided to exploit an adeno‐associated virus (AAV) vector to express influenza antigens to elicit protective antibody responses. The selection of AAV as vector offers several advantages. AAV cannot replicate in humans, and data indicates they do not induce any pathophysiology. They are easy to produce under Good Manufacturing Practice standards and can be re‐administered if needed. Of note, AAV has been already tested to express influenza antigens and showed to confer protection (Lin *et al*, 2009; Sipo *et al*, 2011; Xin *et al*, 2001).

Demminger and colleagues developed AAV vectors expressing wild‐type HA, the surface exposed neuraminidase (NA), or chimeric HA (cHA) containing head regions from influenza A virus subtypes, or HA headless constructs. Constructs were based on proteins encoded by the pandemic influenza virus A/California/7/2009 (H1N1) pdm (Cal/7/9). Control assays confirmed the robust expression of all antigens.

Next, they assessed the immunogenicity in mice challenged intranasally testing side‐by‐side empty AAV vector (expressing GFP) and whole‐inactivated virus (WIV) as controls. As expected, all AAV constructs expressing influenza antigens did elicit reactive antibodies that correlated with the total serum IgG titers. Interestingly, AAV vectors induced antibodies against a panel of different influenza viruses. Somewhat unexpectedly, WIV vaccination also elicited broadly reactive antibodies. Interestingly, neutralizing antibodies were found only in the sera of mice challenged with AAV containing HA, or cHA, and these antibodies were specific for the virus used for prime immunization. Of significant therapeutic relevance, authors demonstrated that AAV vector vaccination induced strong Fc‐activating antibody response against Cal/7/9 but also against the mouse‐adapted heterologous influenza virus PR8.

These positive results encouraged authors to test whether AAV vectors do protect mice from homologous (Cal/7/9) and heterologous (PR8) challenges. Although the mouse model has its limitations in terms of translational potential, it offers the opportunity to obtain first proof‐of‐concept information. As read‐outs of infection, Demminger and co‐workers used weight loss and survival of mice over a 14‐day period. Mice vaccinated with the AAV construct were protected, and preliminary data suggest that three doses of the vaccine were better than two doses. Although these findings are sound, it is important to note that determination of viral titers in the tissues over time should be determined to solidify the read‐outs based on animal behavior, clinical parameters, and/or survival. Some control experiments revealed a reduction in viral titers in vaccinated animals.

In a remarkable tour de force, authors went on to assess the protective efficacy of the AAV vectors in ferrets. This animal model reproduces very closely the course of human influenza. In these experiments, authors also introduce a group immunized with a commercial quadrivalent influenza vaccine of season 2017/18. Animals were vaccinated intranasally three times in 4‐week interval whereas the control group immunized with the commercial vaccine were given two doses by the intramuscular route. It can be argued that the different experimental regime of the groups is not suitable for a comparison between the different vaccine formulations. However, the aim of this work was to demonstrate whether the AAV vectors do confer protection and not whether they are better than the commercial vaccine. In a 3‐day infection protocol, ferrets vaccinated with the AAV vectors showed clear signs of recovery assessing serous nasal exudate, congestion, frequent sneezing, wheezing, and depression. Notably, only in those ferrets vaccinated with the AAV‐HA construct and the commercial vaccine was there a reduction in the load of virus although no sterile immunity was induced. Virus clearance was associated with a significant reduction in pathophysiology and induction of neutralizing antibodies.

This elegant study follows a comprehensive design from in vitro characterization of the AAV vectors to animal pre‐clinical studies probing two different research models, one of them of significant translational potential (Fig 1). The use of research models approximating human disease, ferrets in this case, should be a golden standard approach in any work focusing on vaccine development. The value of the mouse model cannot be disregarded but, certainly, the time is ripe to probe models with increasing translational value.

**Figure 1:**
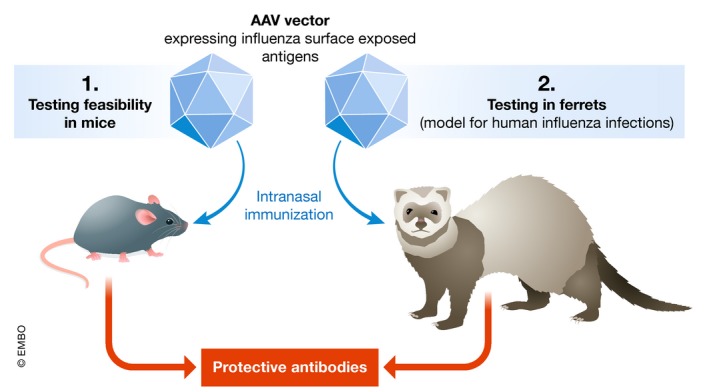
Schematic of the research approach followed to develop AAV vectors as vaccine candidates against influenza After developing AAV vectors expressing influenza surface exposed antigens, authors followed an elegant pipeline probing their vectors first in mice and subsequently in ferrets, research model that accurately recapitulates human influenza infections. Vaccines were administered via the intranasal route, key to develop protective antibodies.

This work also reinforces the utility of AAV vectors as platform to develop vaccines. AAV vectors are already approved by the EMA and FDA for its use in humans and have been tested in pre‐clinical immunization studies against infectious diseases. Demminger and colleagues demonstrate that they should be considered as a valid platform for a broadly protective influenza vaccine. I anticipate that this comprehensive work will lead to significant advances in our quest to develop effective influenza vaccines and may result in similar research to tackle other infections.

I will encourage the vaccinology field to consider and set clear parameters that should be determined in any research like the one presented in this issue of *EMBO Molecular Medicine* (Demminger *et al*, 2020). From clear guidelines on power calculation for the in vivo work to the biological read‐outs that should be analyzed. Among others, I will urge colleagues to consider essential a comprehensive immune profiling at the relevant mucosae coupled with the quantification of cytokines and chemokines. Technology such as mass cytometry could improve significantly the breath of data obtained while, perhaps, uncovering new biology in terms of immune populations relevant for vaccine development.
